# β-Glucan Enhances the Biocontrol Efficacy of Marine Yeast *Scheffersomyeces spartinae* W9 against *Botrytis cinerea* in Strawberries

**DOI:** 10.3390/jof9040474

**Published:** 2023-04-15

**Authors:** Xueyan Chen, Yingying Wei, Xiurong Zou, Zichang Zhao, Shu Jiang, Yi Chen, Feng Xu, Xingfeng Shao

**Affiliations:** 1 State Key Laboratory for Managing Biotic and Chemical Threats to the Quality and Safety of Agro-Products, Zhejiang-Malaysia Joint Research Laboratory for Agricultural Product Processing and Nutrition, College of Food and Pharmaceutical Sciences, Ningbo University, Ningbo 315800, China; 2 Henry Fok School of Food Science and Engineering, Shaoguan University, Shaoguan 512005, China

**Keywords:** biological control, marine yeast, β-glucan, gray mold, strawberry

## Abstract

The marine yeast *Scheffersomyeces spartinae* W9 is a promising biocontrol agent for gray mold caused by *Botrytis cinerea* in strawberries. Improving the biocontrol efficacy of *S. spartinae* W9 is necessary for its commercial application. In this study, different concentrations of β-glucan were added to the culture medium to evaluate its effect on the biocontrol efficacy of *S. spartinae* W9. The results showed that 0.1% β-glucan could increase the biocontrol effect of *S. spartinae* W9 against *B. cinerea* in strawberries and in vitro. We found that adding 0.1% β-glucan to the culture medium promoted the growth of *S. spartinae* W9 in wounds of strawberries, enhanced biofilm formation ability, and secreted more β-1,3-glucanase. In addition, 0.1% β-glucan increased the survival rate of *S. spartinae* W9 under oxidative, thermal, osmotic, and plasma membrane stressors. Transcriptome analysis revealed 188 differential expressed genes in *S. spartinae* W9 cultured with or without 0.1% β-glucan, including 120 upregulated and 68 downregulated genes. The upregulated genes were associated with stress response, cell wall formation, energy production, growth, and reproduction. Thus, culturing with 0.1% β-glucan is an effective way to improve the biocontrol ability of *S. spartinae* W9 against gray mold in strawberries.

## 1. Introduction

Gray mold caused by *Botrytis cinerea* is the primary fungal disease of strawberry fruit during planting and postharvest storage [1]. Chemical fungicides have been used as the most effective method to control gray mold for decades. However, frequent use of chemical fungicides has increased the resistance of fungal pathogens and has had a negative influence on human health and environmental safety [2,3]. In recent years, researchers have been actively seeking alternatives to chemical fungicides. Biological control using microbial agents is considered to be a promising candidate in the management of postharvest diseases. Antagonistic yeasts are widely used to control postharvest diseases because of their biosafety, well-defined mechanism of action, and good commercial potential [4,5,6]. A variety of antagonistic yeasts have been reported to control gray mold decay of fruits, including *Pichia guilliermondii* [7], *Wickerhamomyces anomalus* [8], *Metschnikowia pulcherrima* [9], *Candida pseudolambica* [10], *Metarhizium anisopliae* [11], *Scheffersomyeces spartinae* [12], and so on. However, antagonistic yeasts have not been able to achieve the effect of traditional chemical fungicides in controlling postharvest diseases of fruit [13]. Therefore, it is important to improve the biocontrol efficacy of antagonistic yeasts.

Adding polysaccharides to the culture media may increase the biocontrol activity of antagonistic yeasts. β-glucan is a natural polysaccharide and the main component of yeast cell walls and has various biological activities [14]. β-glucan has been reported to improve various stress tolerances and biocontrol efficacy of *Cryptococcus laurentii* (*Papiliotrema laurentii*) [15,16] and enhance the biocontrol efficacy of *Cryptococcus podzolicus* (*Saitozyma podzolica*) [16] against postharvest decay of apples [17] and pears [18] by upregulating the genes related to cell wall synthesis and increasing its tolerance to oxidative stress. *S. spartinae* W9 is an antagonistic yeast isolated from the intertidal zone marine sediment by our laboratory and a promising biocontrol agent for gray mold in strawberries [12]. To promote the commercial application process, improving the biocontrol efficacy of *S. spartinae* W9 is urgent. Whether β-glucan can enhance the biocontrol efficacy of marine yeast *S. spartinae* W9 has yet to be studied.

Therefore, this study aimed to investigate the effect of β-glucan on the biocontrol efficacy of marine yeast *S. spartinae* W9 and reveal its possible mechanism. In this study, we investigated (1) the effect of different concentrations of β-glucan on antagonistic activities of *S. spartinae* W9 against *B. cinerea* in vitro and in strawberry fruit, (2) the effect of β-glucan on the colonization ability and biofilm forming ability of *S. spartinae* W9, (3) the effect of β-glucan on extracellular hydrolase activity of *S. spartinae* W9, (4) the effect of β-glucan on various forms of stress tolerance of *S. spartinae* W9, and (5) the effect of β-glucan on the transcriptome of *S. spartinae* W9.

## 2. Materials and Methods

### 2.1. Antagonistic Yeast and Fungal Pathogen

The antagonistic yeast *S. spartinae* W9 was preserved in our laboratory and had previously been isolated from the marine sediment in the South China Sea [12]. Consistent with the method used by Wang et al. (2018) and Zhao et al. (2020) [17,18], the activated yeast was suspended in NYDB (nutrient yeast dextrose broth medium) or NYDB supplemented with 0.1%, 0.5%, 1%, and 2% β-glucan (*w/v*), and incubated in an oscillating incubator at 28 °C for 24 h. 

*B. cinerea* was isolated from infected strawberry fruit and preserved in our laboratory. The strain was maintained and propagated on PDA (potato dextrose agar medium). Spore suspensions were washed from the mycelium, cultured for 7 d, and adjusted to 1 × 10^5^ spores mL/L using a hemocytometer.

### 2.2. Fruit

Commercially mature strawberries (*Fragaria ananassa* Duch. cv. Zhangji) were harvested from a greenhouse in the Meishan District, Ningbo, China. Strawberries with uniform color and size and absence of visual injury or lesion were selected for experiments. The strawberries were wiped with 75% ethanol (*v*/*v*) for disinfection and air-dried at 20 °C [19].

### 2.3. Biocontrol Efficacy of S. spartinae W9 Cultured with Different Concentrations of β-Glucan against B. cinerea in Strawberries

This experiment was conducted according to the method described by Fu et al. (2015) [15]. A uniform wound was made at the strawberry equator with a sterilized nail (3 mm deep × 3 mm wide), and each wound was inoculated with 10 µL of any one of the following solutions: (1) sterile water (control), (2) *S. spartinae* W9 cell suspension cultured in NYDB medium (1 × 10^8^ cells/mL), or (3) *S. spartinae* W9 cell suspension cultured in NYDB medium with 0.1%, 0.5%, 1%, and 2% β-glucan (*w/v*) (1 × 10^8^ cells/mL). After two hours, each wound was injected with 10 µL of *B. cinerea* spore suspension (1 × 10^5^ spores/mL). Then all strawberries were stored in a thermostatic chamber at 20 °C with 90% relative humidity. The incidence of gray mold and the diameter of each lesion were observed every day. Every treatment was replicated three times, with 20 fruits selected at random in each replicate. 

### 2.4. Antagonistic Activity of S. spartinae W9 Cultured with β-Glucan against B. cinerea In Vitro

For the mycelia growth test, 100 µL of *S. spartinae* W9 suspensions (cultured in NYDB and NYDB with 0.1%, 0.5%, 1%, and 2% β-glucan) at a concentration of 1 × 10^5^ cells/mL was spread into the PDA medium. A total of 100 µL of sterile water was spread to the PDA and used as the control. Afterwards, a 9-mm diameter plug taken from the edge of the actively growing *B. cinerea* colony was inoculated into the center of each PDA plate. The plates were incubated at 28 °C for 5 d, and the mycelia diameters were measured daily. The experiment consisted of three replicates, with 5 plates per replicate. 

For the spore germination test, cell suspensions of 1 × 10^9^ cells/mL *S. spartinae* W9 (cultured in NYDB and NYDB with 0.1%, 0.5%, 1%, and 2% β-glucan) were prepared. Then 200 µL of yeast suspension was mixed with 200 µL of *B. cinerea* (1 × 10^6^ spores/mL) spore suspension and added into a 1.6-µL PDB medium. An amount of 200 µL sterile water mixed with 200 µL *B. cinerea* spore suspension was used as the control. The spores were incubated for 6 h at 28 °C and 180 rpm on a shaker. The germination rate of *B. cinerea* spores were counted by light microscopy and hemocytometer. At least 100 spores were observed in each treatment, with 3 replicates per treatment.

### 2.5. Population of Yeasts in Strawberry Wounds

In accordance with the method of Zou et al. (2022) [10], strawberries were wounded as described in 2.3 and then inoculated with *S. spartinae* W9 or 0.1% β-glucan-treated *S. spartinae* W9. All strawberries were stored at 20 °C with 90% relative humidity. The amount of yeast at the strawberry fruit wounds was measured at 0 h, 12 h, 24 h, 48 h, and 72 h. The fruit wound tissue was removed with a sterile blade and homogenized in a mortar containing 10 mL of sterile water. A total of 100 µL of the homogenate diluted to the appropriate concentration was spread on NYDA and incubated at 28 °C for 48 h to count the number of colonies. The number of yeast colonies at each wound was expressed as log_10_CFU/wound. The experiment consisted of three replicates, with three strawberries per replicate.

### 2.6. The Effect of 0.1% β-Glucan on the Growth of S. spartinae W9

*S. spartinae* W9 cells (1 × 10^8^ cells/mL) were cultured in NYDB or NYDB with 0.1% β-glucan at 28 °C. The growth of *S. spartinae* W9 was recorded by measuring the absorbance at 600 nm at 12 h, 24 h, 36 h, and 48 h. Three biological replicates were available for each treatment.

### 2.7. Determination of Biofilm Formation

The biofilm formation of *S. spartinae* W9 was assessed using the method of Qiu et al. (2022) [20] with some modifications. An amount of 100 μL of *S. spartinae* W9 suspension (1 × 10^7^ cells/mL) was pipetted into 50 mL of YNB medium and cultured for about 12 h. Yeast precipitate was obtained by centrifugation at 8000 rpm, 4 °C, for 5 min and was washed twice to adjust the concentration of yeast to 1 × 10^8^ cells/mL. Equal amounts of yeast suspension were injected into YNB or YNB containing 0.1% β-glucan and then were incubated at 28 °C, 75 rpm for 12, 18, 24, 36, and 48 h. For biofilm assay, the culture solutions were washed twice with phosphate buffer and treated with methanol for 15 min. Then, 200 μL of 0.4% crystalline violet was added for staining, and after 40 min, the excess dye was removed and washed with sterile water. Lastly, 200 μL of 33% glacial acetic acid was added and incubated for 30 min, and the supernatant was used to measure the absorbance at 590 nm.

### 2.8. Measurement of Extracellular Hydrolases

The ability of *S. spartinae* W9 to produce extracellular hydrolases, mainly including chitinase and β-1,3-glucanase (GLU), was determined by an extracellular hydrolase assay plate [21]. Chitinase and CLU can decompose the colloidal chitin and laminarin, respectively. If *S. spartinae* W9 secreted chitinase and CLU, the hyaline rings would appear in the plates.

According to the result of an extracellular hydrolase assay plate, we determined the GLU activity of *S. spartinae* W9. *S. spartinae* W9 cultured in NYDB or NYDB with 0.1% β-glucan were sampled at 12, 16, 20, 24, 36, and 48 h. The fermentation broth was centrifuged at 6000 rpm for 10 min, and the supernatant was filtered through a sterile membrane for enzyme assay. The GLU activity was determined using the 3, 5-dinitrosalicylic acid method [22], and the absorbance at 540 nm was measured to calculate GLU activity. One unit (U) of GLU was defined as the amount of the enzyme required to produce 1 nmol of glucose per minute, and the results were expressed as U/mL. Each group contained three independent replicates. 

### 2.9. Stress Resistance Assays of S. spartinae W9

As described by Huang et al. (2021) [23], high temperature, NaCl, H_2_O_2_, and sodium dodecyl sulfate (SDS) were used to simulate stress. *S. spartinae* W9 were cultured in NYDB or NYDB with 0.1% β-glucan for 24 h, and the yeast suspensions (1 × 10^8^ cells/mL) were prepared. 

To determine the tolerance of *S. spartinae* W9 to high temperatures, the yeast suspensions were placed into a water bath at 40 °C, 45 °C, and 50 °C for 30 min. The heat-treated yeast suspensions were cooled at room temperature for 10 min and diluted to the appropriate concentration. Then, 0.1 mL of each yeast suspension was spread on a NYDA plate to calculate the survival rate. 

*S. spartinae* W9 or β-glucan-treated *S. spartinae* W9 yeast suspensions were injected into NYDB (control) and a selective medium (NYDB-4% NaCl, NYDB-7% NaCl, NYDB-10% NaCl, NYDB-1.25mM H_2_O_2_, NYDB-2.5mM H_2_O_2_, NYDB-5mM H_2_O_2_, NYDB-10mM H_2_O_2_, NYDB-0.1‰ SDS, NYDB-0.5‰ SDS, NYDB-1‰ SDS). Yeasts were incubated at 28 °C and 180 rpm for 24 h and then diluted to the appropriate concentration to spread on NYDA plates. All plates were incubated at 28 °C for 48 h, and the numbers of yeast colony were recorded. The survival rates of *S. spartinae* W9 or β-glucan-treated *S. spartinae* W9 without stress treatment were used as respective controls. Each treatment contained three independent replicates.

### 2.10. Transcriptomic Analysis

RNA from *S. spartinae* W9 cultured in NYDB with or without 0.1% β-glucan was isolated using TRIzol (Thermofisher, MA, USA) according to the manufacturer’s instructions. The mRNA with PolyA (polyadenylation) was specifically captured and fragmented with a magnesium ion interruption kit (NEBNext^R^ Magnesium RNA Fragmentation Module, cat. E6150S, New England Biolabs, MA, USA). The fragmented RNA was synthesized into cDNA. Then, double-stranded DNA were synthesized and repaired. The fragment sizes were screened and purified using magnetic beads, and the cDNA library was enriched with PCR. RNA-seq was performed by LC-Bio Technology CO., Ltd. Hangzhou, China. Genes with a fold change in expression level ≥ 1.2 and adjusted *p* < 0.01 were identified as differentially expressed genes (DEGs). The DEGs’ data was analyzed for GO and KEGG enrichment analysis. 

### 2.11. Real-Time Quantitative PCR

RNA extracted from *S. spartinae* W9 cultured in NYDB with or without 0.1% β-glucan were first reverse transcribed to cDNA. RT-qPCR was performed using ChamQ Universal SYBR qPCR Master Mix (Vazyme, Nanjing, China). Twelve DEGs were randomly selected for validation by qPCR, and *ACT1* was used as an internal reference gene. Relative gene expression was calculated according to the 2^−ΔΔCt^ method. Table 1 contains a list of the selected genes and the specific primers.

### 2.12. Statistical Analysis

SPSS statistics software version 20 was used to analyze the data. When the number of comparisons within the group was three or more, the data were compared with the mean by implementing Duncan’s multiple analysis using ANOVA; when the number of comparisons within the group was two, the mean was compared using the independent samples *t*-test. Significance was assessed when *p* < 0.05. Data were expressed as mean ± standard deviation.

## 3. Results

### 3.1. Biocontrol Efficacy of S. spartinae W9 Cultured with β-Glucan against B. cinerea in Strawberries

**Figure 1:**
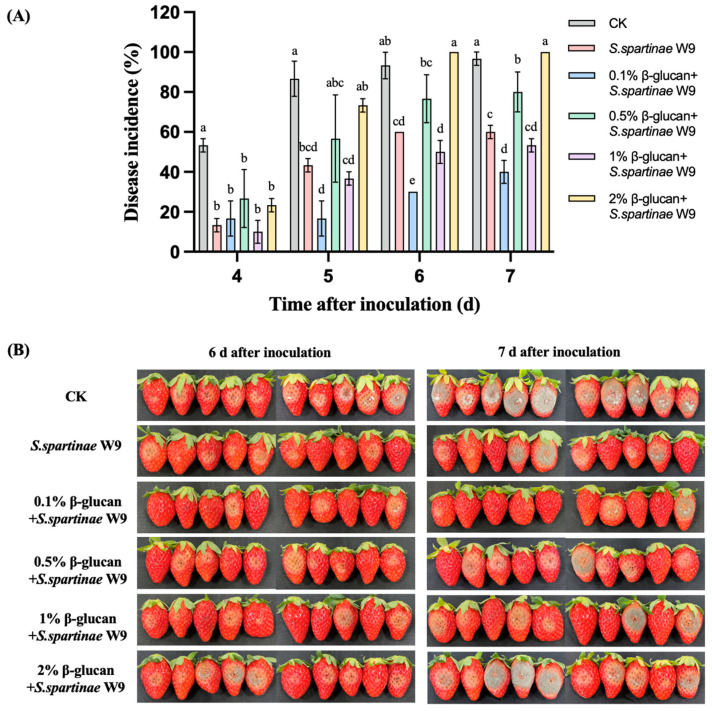
Biocontrol efficacy of *S. spartinae* W9 cultured with different concentrations of β-glucan against *B. cinerea* in strawberries. (**A**) Disease incidence of gray mold in strawberries after the treatment with *S. spartinae* W9 cultured with different concentrations (0, 0.1%, 0.5%, 1%, and 2%) of β-glucan during storage. (**B**) Phenomenon of gray mold in strawberries in each group at day 6 and day 7 after inoculation with *B. cinerea*. Different lowercase letters indicate significant differences between different treatment groups according to Duncan’s test (*p* < 0.05).

As shown in Figure 1A, gray mold decay of strawberries in all groups was observed at the fourth day after inoculation, and *S. spartinae* W9 cultured with or without β-glucan reduced the disease incidence of strawberries. *S. spartinae* W9 cultured with 0.1% β-glucan significantly reduced the disease incidence when compared to yeast without β-glucan after the fourth day (*p* < 0.05). At the end of storage, the disease incidence was 96.7% in the control group, 63.3% in the *S. spartinae* W9 group, and only 40% in the 0.1% β-glucan-treated *S. spartinae* W9 group (Figure 1A,B). Adding 0.1% β-glucan into NYDB effectively improved the biocontrol efficacy of *S. spartinae* W9. 

### 3.2. In Vitro Inhibition of S. spartinae W9 Cultured with β-Glucan against B. cinerea

**Figure 2:**
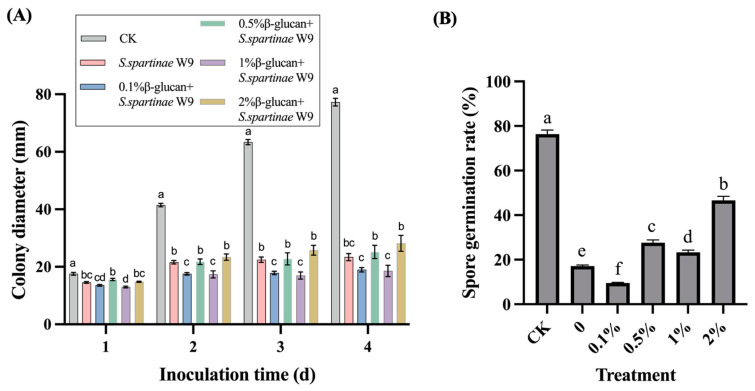
In vitro inhibition of *S. spartinae* W9 cultured with different concentrations of β-glucan against *B. cinerea.* (**A**) Mycelia diameter of *B. cinere* after treatment with *S. spartinae* W9 cultured with different concentrations (0, 0.1%, 0.5%, 1%, and 2%) of β-glucan. (**B**) Spore germination rate of *B. cinerea* after treatment with *S. spartinae* W9 cultured with different concentrations (0, 0.1%, 0.5%, 1%, and 2%) of β-glucan. Different lowercase letters indicated significant differences between different treatment groups according to Duncan’s test (*p* < 0.05).

As shown in Figure 2A, β-glucan enhanced the antagonistic effect of *S. spartinae* W9 against *B. cinerea* in vitro. On the fourth day, the mycelia of *B. cinerea* in the control group covered almost the whole plate, while *B. cinerea* mycelia in the other groups were significantly inhibited (*p* < 0.05). Compared to the *S. spartinae* W9 cultured without β-glucan, 0.1% and 1% β-glucan were effective in increasing the inhibition of *B. cinerea* mycelial growth, for which the diameter of *B. cinerea* plaques in the *S. spartinae* W9 treatment group was 23.4 mm, while the diameters of the 0.1% and 1% β-glucan-induced *S. spartinae* W9 treated groups were 18.8 mm and 18 mm, respectively. 

Figure 2B shows that *S. spartinae* W9 cultured with or without β-glucan significantly inhibited spore germination of *B. cinerea* (*p* < 0.05). *S. spartinae* W9 cultured with different concentrations of glucan showed different inhibitory effects on spore germination of *B. cinerea*. The germination rate of *B. cinerea* spores in 0.1% β-glucan-treated *S. spartinae* W9 was 9.5%, which was significantly lower than the 17.1% in the *S. spartinae* W9 group (*p* < 0.05). Only 0.1% β-glucan increased the ability of *S. spartinae* W9 to inhibit spore germination of *B. cinerea*.

### 3.3. Effects of 0.1% β-Glucan on the Growth of S. spartinae W9 In Vitro

*S. spartinae* W9 yeasts were cultured in NYDB and NYDB containing 0.1% β-glucan at a concentration of 0.1% for 48 h, respectively. The OD_600_ of *S. spartinae* W9 in both media showed no significant difference (*p* > 0.05; Table 2). The results indicated that 0.1% β-glucan had no significant effect on the growth of *S. spartinae* W9 in vitro.

### 3.4. Effects of 0.1% β-Glucan on Colonization Ability in Strawberries and Biofilm Forming Ability of S. spartinae W9

**Figure 3:**
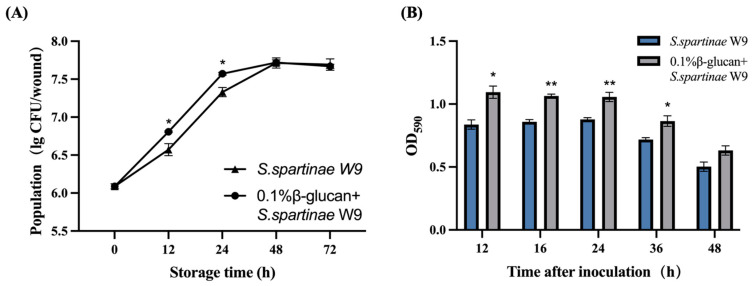
Effects of 0.1% β-glucan on colonization ability in strawberries and biofilm forming ability of *S. spartinae* W9. (**A**) Population dynamics of *S. spartinae* W9 cultured in NYDB or NYDB with 0.1% β-glucan in wounds of strawberries at 20 °C. (**B**) Biofilm forming ability of *S. spartinae* W9 cultured with or without 0.1% β-glucan stained with crystal violet. * indicate a significant difference between *S. spartinae* W9 cultured with or without 0.1% β-glucan identified by the *t*-test (*, *p* < 0.05; **, *p* < 0.01).

*S. spartinae* W9 cultured with or without β-glucan was able to grow rapidly in the wounds of strawberries, and its population levels reached the highest value at 24 h (Figure 3A). The population of the β-glucan-induced *S. spartinae* W9 increased from 6.1 log_10_ CFU wound^−1^ to 7.7 log_10_ CFU wound^−1^ at 24 h, which was significantly higher than that of *S. spartinae* W9 cultured in NYDB (*p* < 0.05). The growth rate of the β-glucan-treated *S. spartinae* W9 within 24 h was higher than *S. spartinae* W9 cultured in NYDB.

Crystal violet staining was used to further evaluate the biofilm formation capacity of *S. spartinae* W9. During the 24 h of yeast culture, the biofilm formation capacity remained stable in each group (Figure 3B). The OD_590_ of *S. spartinae* W9 cultured with β-glucan was consistently higher than that of *S. spartinae* W9 cultured without β-glucan. This indicated that β-glucan promoted the formation of biofilm for *S. spartinae* W9 and enhanced its biofilm formation capacity. 

### 3.5. Effects of 0.1% β-Glucan on Extracellular Hydrolase of S. spartinae W9

**Figure 4:**
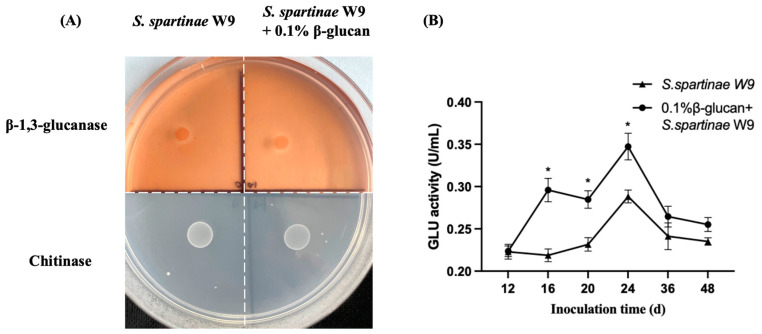
Effects of 0.1% β-glucan on extracellular hydrolase activity of *S. spartinae* W9. (**A**) Results of the extracellular hydrolase assay plates of *S. spartinae* W9 cultured with or without 0.1% β-glucan. (**B**) GLU activity of *S. spartinae* W9 cultured in NYDB or NYDB containing 0.1% β-glucan. * indicate a significant difference between *S. spartinae* W9 cultured with or without 0.1% β-glucan identified by the *t*-test (*p* < 0.05).

The extracellular hydrolase assay plates showed that *S. spartinae* W9 could secrete GLU but not chitinase (Figure 4A). The 0.1% β-glucan could not induce *S. spartinae* W9 to secrete chitinase. The extracellular GLU activities of *S. spartinae* W9 cultured in NYDB with 0.1% β-glucan were significantly higher than those of *S. spartinae* W9 cultured in NYDB during the culture period (Figure 4B), indicating that β-glucan could induce *S. spartinae* W9 to secrete more GLU.

### 3.6. 0.1% β-Glucan Increased the Survival rate of S. spartinae W9 under Different Stresses

**Figure 5:**
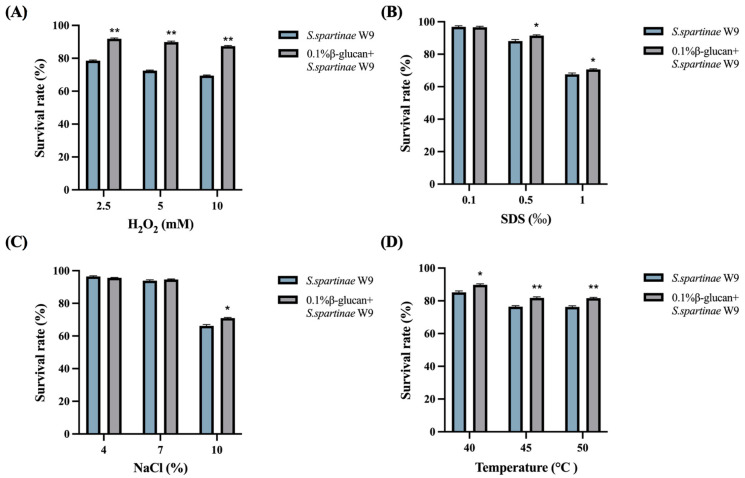
Effects of 0.1% β-glucan on the survival rate of *S. spartinae* W9 under different stresses. (**A**) The survival rate of *S. spartinae* W9 under oxidative stress caused by H_2_O_2_. (**B**) The survival rate of *S. spartinae* W9 under plasma membrane stress caused by SDS. (**C**) The survival rate of *S. spartinae* W9 under hyperosmotic stress caused by NaCl. (**D**) The survival rate of *S. spartinae* W9 under high temperature stressor. Asterisks indicate a significant difference between *S. spartinae* W9 cultured with or without 0.1% β-glucan identified by the *t*-test (*, *p* < 0.05; **, *p* < 0.01).

*S. spartinae* W9 cells harvested from NYDB with 0.1% β-glucan showed higher survival rates under oxidative, thermal, osmotic, and plasma membrane stresses, compared with the yeast cells harvested from NYDB (Figure 5). The survival rate of *S. spartinae* W9 under 10 mM H_2_O_2_, 10% NaCl, 1% SDS, and 50 °C were 66.2%, 68.2% 67.7%, and 76.3%, respectively, while those of β-glucan-treated *S. spartinae* W9 were 87.4%, 71.0%, 70.6%, and 81.63%, respectively (Figure 5). The 0.1% β-glucan increased the survival rate of *S. spartinae* W9 under the 10 mM H_2_O_2_ environment by 32% compared to the control and significantly improved its tolerance to oxidative stress.

### 3.7. RNA-seq Analysis and RT-qPCR Validation

A total of 40.87 G clean reads were obtained from *S. spartinae* W9 cultured in NYDB with or without β-glucan. The Q30 levels were both higher than 70%, indicating that the base quality was up to standard. GC contents were both above 40%, and the percentage of valid reads exceeded 95%, which indicated that the data were reliable. By comparing the gene expression levels between *S. spartinae* W9 cultured in NYDB with and without β-glucan, 188 genes were identified as DEGs, including 120 upregulated genes and 68 downregulated genes. 

**Figure 6:**
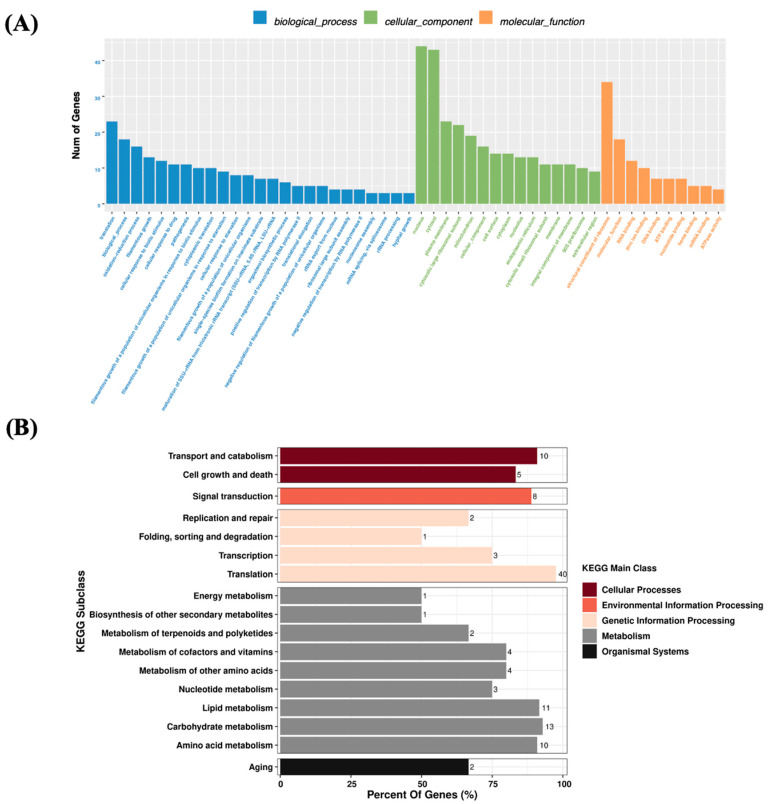
GO (**A**) and KEGG (**B**) enrichment analysis of DEGs in *S. spartinae* W9 cultured with and without 0.1% β-glucan. The numbers behind the bars stand for the number of genes involved in the metabolism.

A total of 172 DEGs were able to be annotated in the GO database and were enriched into three major categories (Figure 6A). In the biological process category, the DEGs were involved in translation, the biological process, the oxidation-reduction process, and so on. The highly enriched DEGs of the cellular components were involved in cell organelles and cell membranes. The subclass with the most enriched DEGs by molecular function was the structural constituent of ribosome (34 DEGs) subclass, followed by RNA binding (12 DEGs). The DEGs were classified into five categories by KEGG enrichment analysis (Figure 6B). Among them, the pathway with the most DEGs was translation, involving 40 genes. The second pathway was the carbohydrate metabolism pathway, with 13 DEGs. 

**Figure 7:**
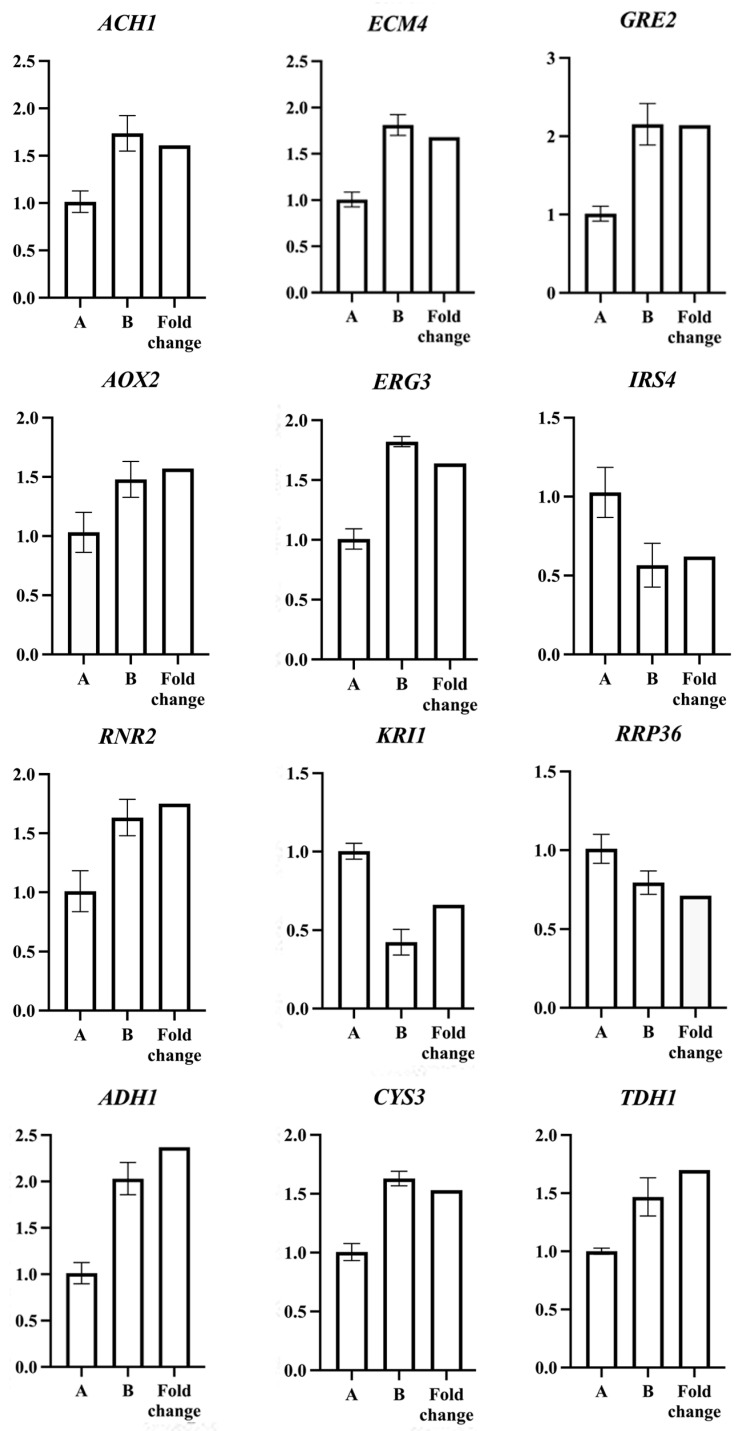
Results of verification of differently expressed genes by RT-qPCR. A: Relative gene expression level of *S. spartinae* W9 cultured in NYDB. B: Relative gene expression level of *S. spartinae* W9 cultured in NYDB with 0.1% β-glucan. Fold Change: Transcriptome sequencing results. Bars represent standard errors.

RT-qPCR was performed on 12 genes to validate the results of RNA-seq. The transcript levels of all selected genes determined by RT-qPCR were consistent with those obtained using RNA-seq (Figure 7).

## 4. Discussion

A bottleneck in the development of antagonistic yeasts has been the limited biocontrol effect in practical application. Therefore, it is very important to enhance the biocontrol efficacy of antagonistic yeasts. It has been reported that β-glucan could improve the biocontrol efficacy of *C. laurentii* and *C. podzolicus* against postharvest blue mold of pears [24]. In the present study, our results also confirmed that β-glucan was an effective elicitor to improve the biocontrol ability of *S. spartinae* W9 on gray mold of strawberry fruit. The 0.1% β-glucan enhanced the biocontrol efficacy and antagonistic activity of *S. spartinae* W9 against *B. cinerea* (Figure 1 and Figure 2). Despite the differences in PDA medium and in fruit [25], the increased antagonistic effects of *S. spartinae* W9 by β-glucan were consistent. Therefore, we selected 0.1% β-glucan to culture *S. spartinae* W9 and reveal the involved mechanism. 

The key factor affecting the biocontrol activity of antagonistic yeasts has been proposed to be the rate of colonization [26]. Yeasts rapidly consume limited nutrients, thereby inhibiting spore germination of fungal pathogens [27]. This study showed that the addition of β-glucan to the medium had no effect on the growth and reproduction of *S. spartinae* W9 in vitro, while β-glucan could promote the colonization of *S. spartinae* W9 in strawberry wounds within 24 h of storage (Figure 3A). Wang et al. (2018) also found that β-glucan increased the growth ability of *C. podzolicus* in apple wounds [28]. Di Francesco et al. (2017) found that the colonization ability of yeast on the wounds has a certain correlation with the biofilm-forming ability [28]. High-density yeast forms exopolysaccharide capsules, which can more firmly adhere to fruit wounds and prevent pathogen infection [29,30]. Our results showed that the addition of 0.1% β-glucan to the culture medium effectively improved the biofilm formation capacity of *S. spartinae* W9 (Figure 3B). β-glucan improved the colonization ability in strawberries and biofilm forming ability of *S. spartinae* W9, which inhibited the growth of *B. cinerea*, thus reducing the incidence of gray mold in strawberries. Our findings are similar to the previous reports, which showed that some exogenous nutrients could induce antagonistic yeasts to enhance the biocontrol efficacy by improving the growth ability at fruit wounds and biofilm formation ability [31,32]. 

The ability of antagonistic yeast to resist pathogens also depends on the secretion of extracellular hydrolase. GLU can hydrolyze glucan, which is the major component of fungal cell walls, leading to cell malformation and cytoplasmic leakage, and is one of the possible mechanisms of biocontrol of antagonistic yeasts [33]. GLU is an inducible hydrolase enzyme, and the presence of β-glucan could induce the secretion of GLU from antagonistic yeasts. Our results showed that *S. spartinae* W9 could secret GLU, and 0.1% β-glucan induced the higher extracellular GLU activities (Figure 4). This suggests that 0.1% β-glucan could induce *S. spartinae* W9 to secrete more GLU, which might be an important factor for the enhanced biocontrol efficacy of *S. spartinae* W9. This finding is consistent with the report of Zhao et al. (2020), which showed that higher GLU activity of *C. podzolicus* is an important factor for the increased biocontrol effect of *C. podzolicus* cultured with β-glucan [17,18].

In practical application environments such as orchards and postharvest storage, various abiotic stressors could decrease the biocontrol efficacy of antagonistic yeasts [34]. Thus, improving the ecological adaptability and resistance of antagonistic yeasts is an effective way to ensure their biocontrol efficacy. SDS is an organic compound that penetrates cell membranes, causes damage to the plasma membrane, and limits cell growth [35,36]. Oxidative adversity was simulated using hydrogen peroxide because a fruit wound is an environment with high levels of reactive oxygen species [37], which cause oxidative damage to the cell membrane component membrane lipids, thereby destroying the integrity of the cell [38]. NaCl is a common osmotic stressor, and high NaCl has the effect of inhibiting cell growth [39]. The present study showed that the growth of *S. spartinae* W9 was significantly inhibited under these stressors, while *S. spartinae* W9 cultured with 0.1% β-glucan could enhance tolerance under these stressors (Figure 5). Fu et al. (2015) also found that 0.5%-β-glucan-induced *C. laurentii* can survive better under heat and oxidative stress [15]. These findings suggest that β-glucan could improve the stress tolerance of antagonistic yeasts and help to improve their biocontrol efficacy in practical applications.

The transcriptome analysis was conducted to explore the molecular mechanism of β-glucan, enhancing the biocontrol efficacy of *S. spartinae* W9. The results showed that β-glucan caused 188 DEGs, and the DEGs were related to cell wall formation and cell integrity, energy production, growth, and reproduction (Figure 6). Some upregulated DEGs are associated with stress response, including *GRE2*, *CYS3*, *AOX2*, and *ERG3*. GRE2 has been reported to be induced in yeast cells in response to oxidative stress [40] and osmotic stress [41]. Similarly, elevated AOX2 implies an acceleration of oxidative processes in mitochondria and limitation of ROS production [42]. The upregulation of the *ERG3* gene due to β-glucan was beneficial for maintaining the stability of yeast cell plasma membranes and improving the resistance of yeast to oxidative stress [43]. *CYS3* can play a protective role for yeast in a state of oxidative stress and promote the efficiency of yeast cell proliferation [44,45,46]. The upregulation of *GRE2*, *CYS3*, *AOX2*, and *ERG3* could explain the increased oxidative stress tolerance of *S. spartinae* W9 cultured with 0.1% β-glucan.

The yeast cell wall serves a variety of biological functions, including protecting against environmental stresses. It also affects the permeability of the cell wall when it is attacked by substances that disrupt cell integrity [47]. The genes *TDH1* and *ECM4* have been shown to be involved in cell wall structure [48,49]. Compared to the control, the higher expression of *TDH1* and *ECM4* in *S. spartinae* W9 cultured with 0.1% β-glucan promoted cell wall biosynthesis and biocontrol efficacy.

Intracellular energy is heavily depleted when yeast cells are under abiotic stress [50]. ACH1 provides acetyl coenzyme A (acetyl-CoA) to mitochondria by transferring the CoA group to acetate, which may accelerate metabolism in the TCA cycle and glycolysis [51]. The gene *ADH1* encodes alcohol dehydrogenase, which is the primary enzyme for the reoxidation of NADH in yeast. Zhong et al. (2021) showed that inactivation of ADH1 caused the accumulation of NADH, resulting in the imbalance of NADH/NAD and growth retardation [52]. The upregulation of *ACH1* and *ADH1* in *S. spartinae* W9 cells cultured with 0.1% β-glucan led to higher colonization ability in strawberry wounds and improved biocontrol efficacy. In addition, upregulation of the *RNR2* gene required for DNA repair may be helpful for maintaining cellular integrity [53]. In brief, β-glucan increased the energy metabolism and cellular integrity of *S. spartinae* W9 cells.

The transcriptome analysis demonstrated that the 0.1 % β-glucan-induced *S. spartinae* W9 achieved better proliferation and enhanced biological activity by altering cellular processes, energy metabolism, and stress responses. Similarly, Zhao et al. (2020) used transcriptomic analysis to reveal that β-glucan accelerated the synthesis of cell walls and energy of *C. podzolicus*, enhanced its antioxidant ability, and thus improved the biological control efficacy [18].

## 5. Conclusions

Adding 0.1% β-glucan into the culture medium significantly enhanced the biocontrol efficacy of *S. spartinae* W9 against *B. cinerea* in strawberries. β-glucan improved the colonization ability of yeast in strawberries, the biofilm forming ability, and the stress tolerance of *S. spartinae* W9. β-glucan also upregulated the genes involved in stress responses, cell wall formation, energy metabolism, growth, and reproduction of *S. spartinae* W9.

## Figures and Tables

**Table 1:** Primers used for RT-qPCR analysis of *S. spartinae* genes.

Gene ID	Gene Name	Forward and Reverse Primers (5′ to 3′)
gene-KQ657_000363	*CYS3*	F: TGGGTGTTTTGGCAACCAAC R: ACCTCTGTGAGCCAACCAAG
gene-KQ657_002550	*GRE2_4*	F: TTTCACATTGCGTCTCCCGT R: GCTGAGGTGCCTTATCCGTT
gene-KQ657_002037	*ERG3*	F: CGGATGGTCTCTTCCACTCC R: CTGTTGCCCATATCGAGGGT
gene-KQ657_001934	*ACH1*	F: CGTTTCTACGCCAACTGGGA R: GGTGGAGTTAGCATGAGCGT
gene-KQ657_000095	*AOX2*	F: CATGCCGCACCTGTTTTAGT R: GGCTCTTTAACCGGTGGTGT
gene-KQ657_001274	*TDH1*	F: ACAAGGACTGGAGAGGTGGT R: TTACCGACAGCCTTAGCAGC
gene-KQ657_000537	*ECM4_1*	F: TTCGCTTCCGACCAAGAGAC R: TGACCACCAGCAGTGTGAAT
gene-KQ657_001686	*KRI1*	F: AGGCCGAGACCATTGAAGTC R: CTGGTGCTTCTTGGTCGCTA
gene-KQ657_001371	*IRS4*	F: GCTACGAAGCTTGTGGGAGT R: GCAGACAATCGAGCAGCAAC
gene-KQ657_001105	*RRP36*	F: ACCTGTTTCGGTAGTCAGGG R: CATCTTGCTCCGTTGCTTGG
gene-KQ657_000758	*ADH1_1*	F: ATGGGTTGCAGTCTCTGGTG R: CCTTCTCTTCGCCACCATCA
gene-KQ657_003270	*RNR2*	F: AGATGCCCTTCCAGTGTCTT R: AGGAGAAAGCATTGGCTGCT
gene-KQ657_004479	*ACT1*	F:CAGACCTGCTGACTTGGGTT R:AGAGGATGGGGCCAACAAAG

**Table 2:** Growth of *S. spartinae* W9 in NYDB or NYDB containing 0.1% β-glucan.

Culture Conditions	OD_600_			
	12 h	24 h	36 h	48 h
NYDB	1.25 ± 0.011 a	2.22 ± 0.006 a	2.42 ± 0.005 a	2.51 ± 0.001 a
NYDB with 0.1%β-glucan	1.27 ± 0.009 a	2.23 ± 0.014 a	2.41 ± 0.007 a	2.51 ± 0.018 a

Same letter in each column means no significant difference according to the independent samples *t*-test (*p* > 0.05).

## Data Availability

The data presented in this study are included in the article. Further inquiries can be directed to the corresponding author.
